# How do illness identity, patient workload and agentic capacity interact to shape patient and caregiver experience? Comparative analysis of lung cancer and chronic obstructive pulmonary disease

**DOI:** 10.1111/hsc.13858

**Published:** 2022-05-28

**Authors:** Kate Lippiett, Alison Richardson, Carl R. May

**Affiliations:** ^1^ School of Health Sciences University of Southampton Southampton UK; ^2^ NIHR Applied Research Collaboration Wessex Southampton UK; ^3^ University Hospital Southampton NHS Foundation Trust Southampton UK; ^4^ Faculty of Public Health and Policy London School of Hygiene and Tropical Medicine London UK; ^5^ NIHR Applied Research Collaboration North Thames London UK

**Keywords:** caregivers, chronic obstructive, health resources, lung neoplasms, pulmonary disease, qualitative research, workload

## Abstract

Some patients have to work hard to manage their illness. When this work outweighs capacity (the resources available to patients to undertake the illness workload and other workloads such as that of daily life), this may result in treatment burden, associated with poor health outcomes for patients. This cross‐sectional, comparative qualitative analysis uses an abductive approach to identify, characterise and explain treatment burden in chronic obstructive pulmonary disease (COPD) and lung cancer. It uses complementary qualitative methods (semi‐structured interviews with patients receiving specialist care *n =* 19, specialist clinicians *n* = 5; non‐participant observation of specialist outpatient consultations in two English hospitals [11 h, 52 min] *n* = 41). The findings underline the importance of the diagnostic process in relation to treatment burden; whether diagnosis is experienced as a biographically disruptive shock (as with lung cancer) or is insidiously biographically erosive (as with COPD).

**What is known about this topic?**

- Treatment burden has been linked to poorer health‐related outcomes for patients.
- People have varying abilities and resources (called capacity) to manage the work of healthcare and daily life.

**What this paper adds?**

- Biographical disruption or erosion after diagnosis impacts on patient work and patient capacity.
- Delegated tasks, particularly those involving health behaviours, may be hard for patients, caregivers and healthcare professionals/systems to see as treatment work.
- Treatment burden is more than workload‐capacity imbalance; it is a complex interrelationship between illness identity, work and capacity mediated through status passages.

## 1 INTRODUCTION

In recent years there has been increased interest in the work of patient‐hood. This built on the germinal research of Strauss, Glaser and colleagues who investigated the social practices through which lived experiences of illness are formed and organised, and through which illness identities are socially constructed (Corbin & Strauss, 1988; Strauss et al., 1985). Recent epidemiological and demographic transitions have meant people are living increasingly with chronic diseases requiring *management* rather than cure (Taylor & Bury, 2007). While attention has recently been drawn to the acute consequences of the COVID‐19 pandemic, these will not displace endemic long‐term conditions. Indeed, there is increasing recognition of ‘long Covid’ where survivors of COVID‐19 live with persistent symptoms, describing themselves as ‘long haulers’ (Kingstone et al., 2020). Latterly, research has focused on patient work as a practical problem, formed around normative expectations healthcare policy and providers have of patients—especially with long‐term conditions—that patients are motivated to *participate in their own care* and this participation will be *effective* (Hunt & May, 2017).

In this article, we define patient and caregiver *work* as the ‘affective, cognitive, informational, material, physical and relational tasks’ (p.1) (Lippiett et al., 2019) going into participation in care, and the wider work of ‘managing’ ill‐health. We are equally interested in patient and caregivers' *capacity* to perform these tasks. We define this capacity as the affective, cognitive, informational, material, physical and relational resources available to them, and, importantly, *which they are able to mobilise* to achieve participation in care and meet normative expectations of healthcare providers. Where this work outweighs capacity of patients and carers, patients may experience treatment burden (Shippee et al., 2012). Systematic reviews and meta‐syntheses of qualitative studies of lived experiences of illness have characterised the multidimensional nature of workload‐capacity interactions and their effects on patients, their caregivers and members of their wider social networks seeking to meet treatment demands (Boehmer et al., 2016; Demain et al., 2015; Gallacher et al., 2013; Jani et al., 2013; Lippiett et al., 2019; Roberti et al., 2018; Sav et al., 2015, 2017). Identification of treatment burden is important because it may lead to poor adherence to medication and treatment regimens with consequent increased hospitalisation, increased mortality and impaired health‐related quality of life (Daker‐White et al., 2018; Eton et al., 2012; Gallacher et al., 2011; May et al., 2009).

In this article, we contrast different experiences of workload and capacity in two conditions: lung cancer and chronic obstructive pulmonary disease (COPD), and further identify, characterise and explain important elements of treatment burden. These two common diseases are non‐communicable, resulting from exposure to tobacco smoke in ~90% of cases (National Institute for Clinical Excellence, 2019). Although recent advances in treatment such as immunotherapies are extending survival (Jones & Baldwin, 2018), in the United Kingdom, lung cancer is still characterised by late diagnosis, relatively rapid disease progression, and low 5‐year survival rates. COPD is a long‐term condition with various therapeutic regimens available, but with only smoking cessation proven to delay disease progression (Jiménez‐Ruiz et al., 2015). Because both diseases are usually smoking‐related, they may carry additional burdens of assumed personal moral culpability for patients, and associated stigma (Rose et al., 2017). Both diseases are normally lethal.

In this study, we built on Burden of Treatment Theory (May et al., 2014), and undertook qualitative systematic reviews comparing and contrasting treatment burden, symptom burden, and disease trajectory in COPD and other long‐term conditions (May et al., 2016; Roberti et al., 2018), and of COPD and lung cancer (Lippiett et al., 2019). From these, we developed a taxonomy of patient experiences of ‘workload’ and ‘capacity’, finding diagnosis (and subsequent illness identity) could initiate significant differences in normative expectations of patient and caregiver behaviour and treatment workload delegated to patients (Lippiett et al., 2019). Second, we drew on Status Passage Theory (Glaser & Strauss, 1971), to explore the micro‐level dynamics of illness identity and associated relations between professionals, patients and caregivers over time. This supported understanding of treatment burden over illness trajectories as a problem of identity formation shaped by feelings of culpability and stigma (Goffman, 1968).

## 2 METHODS

We used two complementary qualitative data collection methods (semi‐structured interviews, non‐participant observation) to facilitate comparison.

We pragmatically selected two southern England hospitals. Specialist respiratory and oncology clinicians in participating sites screened potential participants attending outpatient clinics during recruitment (December 2017–August 2018). Patient participants were English speakers, ≥18, diagnosed with either lung cancer or COPD, under oncology or respiratory services. Patients were excluded if deemed unfit to participate owing to their medical condition or could not provide informed consent. Maximum variation sampling (Palinkas et al., 2015) ensured a range of age, sex, living situation, employment status, disease stage and treatment regimen. Clinician participants worked in specialist respiratory/oncology services in participating sites and were present at observed consultations. KAL obtained informed written consent from patients and clinicians interviewed/observed; verbal consent from friends/family members present during observations/interview.

KAL undertook semi‐structured interviews (23–63 min) with patients at venues of their choice (in homes in all but one case). Interviews focused on diagnosis (illness identity), workload and capacity. KAL also undertook observations of consultations in hospitals (5–52 min, totalling 11 h, 52 min), supported by an observation record guide. Interviews and observations were transcribed verbatim and anonymised. Participants could comment on interview transcripts to ensure they accurately reflected interviews. KAL took field notes to capture immediate insights and consider data collection reflexively. Our coding framework which KAL used to line‐by‐line code observations/interviews is included in online supplementary documentation (Appendix S1). We took an abductive approach to data analysis, exploring variation through grouping‐related codes into datasets (conditions; perspectives of patients, family members and clinicians), at different points in illness trajectories and in different situations (e.g. treatment workload in hospital vs. home) (Tavory & Timmermans, 2014). We met often throughout data collection and analysis to discuss findings and to think reflexively about KAL's assumptions as the primary researcher. The arc of abductive interpretation of these qualitative data led to a complex array of constructs (see Appendix S2).

Research Ethics Committee approval was granted by NHS (England) South West. REC reference: 17/SW/0162.

## 3 RESULTS

KAL interviewed 19 patients: 10 with COPD, nine with lung cancer. She observed 41 outpatient consultations with 41 patients, five clinicians: three respiratory clinicians (1 doctor, 2 nurses) and two lung cancer clinicians (1 doctor, 1 nurse). Table 3 sets out conventions for describing participants. Table 1 describes patient participants' characteristics. In Table 2, we identify and characterise specific activities constituting patient work.

**TABLE 1:** Characteristics of patient participants

Characteristics	Condition
Lung cancer *n* = 26	Non‐small cell lung cancer Stage 2b *n* = 1
COPD *n* = 34	Non‐small cell lung cancer Stage 3a *n* = 2
	Non‐small cell lung cancer Stage 3b *n* = 1
	Non‐small cell lung cancer Stage 3c *n* = 1
Sex	Non‐small cell lung cancer Stage 4 *n* = 9
Female *n* = 27	Small cell lung cancer Stage 3b *n* = 1
Male *n* = 33	Small cell lung cancer Stage 4 *n* = 1
	Mesothelioma Stage 2 *n* = 1
Age	No access to clinical notes, stage unknown *n* = 19 (patients interviewed)
Mean = 70	Mild COPD *n* = 3
Range 41–88	Moderate COPD *n* = 6
	Severe COPD *n* = 9
	Very severe COPD *n* = 2
	Stage of COPD not documented *n* = 4
Treatment regimens: (N.B. patients may be on more than one regimen so number > 60)	Chemotherapy *n* = 10
	Radiotherapy *n* = 4
	Surgery *n* = 1
	Immunotherapy *n* = 9
	EGFR‐TK inhibitor *n* = 4
	Active surveillance (mesothelioma) = 2
	Pulmonary rehabilitation *n* = 14
	Smoking cessation *n* = 4
	Weight management *n* = 2
	Inhalers *n* = 14
	Nebulisers *n* = 2
	Oxygen *n* = 7
	Anti‐inflammatory macrolide *n* = 8
	Standby antibiotics ‘rescue pack’ *n* = 8

**TABLE 2:** The hard work of being sick: Comparison of lung cancer and COPD

Lung cancer	Task sets	COPD
Attend appointments with lung cancer surgeon; oncology healthcare professional; general practitioner (GP); professionals dealing with comorbidities. Undergo blood and urine test(s); undergo scans, biopsies, bronchoscopies. Undergo treatment (radiotherapy, immunotherapy, chemotherapy, surgery). Participate in research (drug trials). Attend hospital for inpatient stay. Make payments for private consultations with health professionals, investigations, and treatments. Pay for hospital parking.	Receiving formal care	Attend appointment with respiratory physician, nurse, and physiotherapist; attend appointment with GP, practice nurse. Attend pulmonary rehabilitation, attend post‐rehabilitation surveillance and care. Undergo scans, blood tests, flu/pneumococcal vaccination, spirometry and breathing tests, electrocardiograms (ECGs) and 6‐minute walk tests, sputum tests, and bronchoscopies. Attend hospital for inpatient stay. Receive home visit from respiratory team (oxygen assessment), respiratory team (supported discharge). Make contact with smoking cessation team. Pay for hospital parking
Seek advice and assistance from oncology team, attend emergency department (ED) out of hours, seek advice from GP (by telephone/in person/home visit)	Help‐seeking	Seek advice and assistance from GP (telephone/in person/home visit); seek advice from specialists/respiratory team members; seek advice from family member. In exacerbation events, take rescue pack; call ambulance; negotiate with ambulance crew; attend ED
Implement breathing techniques. Monitor for fits and infection. Undergo treatment medications (chemotherapy; tyrosine kinase inhibitors). Collect and self‐administer medications (steroids, antibiotic, painkillers, anti‐emetics, anti‐seizure medication). Have injection (monoclonal antibodies), Undergo blood boosting injections	Managing symptoms and medications	Monitor peak flow, oxygen saturations, self for infection. Implement breathing techniques, weight/diet management, increase/maintain physical activity, smoking cessation (patient to cease). Take medication (diuretic, nebulisers, painkillers, steroids, anti‐inflammatory macrolides, antibiotics, anti‐hypertensives, beta‐blockers, statin, inhalers, and diabetic medication). Titrate medication according to symptoms. Use oxygen. Restock rescue pack. Go to chemist (for inhaler preparation). Attend breathlessness classes and Breathe easy (peer support groups). Attend pulmonary rehabilitation and follow‐up.
Learn about condition, symptom and disease progression; learn about breathing techniques; learn about and discuss range of treatments (surgery, chemotherapy, radiotherapy, immunotherapy; discuss treatment options (continuing with tyrosine kinase inhibitor); discuss treatment options (immunotherapy) and tyrosine kinase inhibitors. Discuss weaning/titrating of medications; pathophysiological side effects of treatment, decide not to accept treatment, decide to discontinue treatment. Have radiotherapy mask fitted Learn about and consent to participate in research (potential new treatment). Find out about complementary therapies. Find out about and claim for welfare benefits (social services). Contact support groups.	Learning about and negotiating conditions and care	Learn about condition, breathing tests, scans, and medications. Learn about treatment, smoking cessation, oxygen, lung surgery, breathing techniques, bi‐level positive airway pressure (BIPAP). Learn about treatment (additional written information given by healthcare professional). Discuss treatment options (long term oxygen therapy, medication, smoking cessation, starting/changing/continuing medication, and lung surgery), breathlessness classes and pulmonary rehabilitation. Explain condition to general public. Teach other people how to manage treatment. Research alternative treatment options. Research Singing for Lung Health
Arrange oxygen	Using medical equipment and devices	Use acapella, Have oxygen delivered, Negotiate with oxygen company re holiday, Purchase oxygen saturation probe, Purchase nebuliser machine

**TABLE 3:** Description of identification method for participants

Participant type	Description of identification method	Example
Patients	Identified by component of the study in which they were participating (OBS for observation and INTS for interview), then PA for patient, then by order in which they were recruited	OBS‐PA‐001, INTS‐PA‐001
Clinicians	Identified by component of the study in which they were participating (OBS for observation and INTS for interview), then CL for clinician, then by order in which they were recruited	OBS‐CL‐001, INTS‐CL‐001

### 3.1 Diagnosis, illness identity and work

In lung cancer, the diagnostic moment was characterised by shock and existential crisis. Patients and family members seemed to understand illness trajectories were likely to be short. Against this background, participants evinced a sense of treatment as hope, a bulwark against the existential threat of cancer. Treatment for lung cancer was a priority for patients and their family members, taking precedence over other claims on their time and energy. Indeed, as illness careers were played out, a focus of clinical encounters was continuing to identify further treatment options, so treatment cessation did not have to be faced:

Lung cancer doctor: So…it's a new treatment…it's different to what you had before….And I suppose the reality is…in the last few months we have been trying to find something to do.

Patient: Yeah, that's right.

Doctor: because we'd run out of options and this was a new option that's been made available to us.

Patient: That's it.

(OBS‐PA‐029)

In contrast to people with lung cancer whose diagnostic moment was inscribed indelibly on their memory, some people living with COPD struggled to pinpoint a moment of diagnosis. Others could not be sure they had ever been offered a formal diagnosis at all. Years could elapse between symptom presentation and confirmation of diagnosis. Unlike cancer, COPD has no clear public narrative (British Lung Foundation, 2018). Indeed, COPD is not a unitary pathological entity at all; it involves several complex, heterogeneous and dynamically interacting processes affecting airways and lungs (Singh et al., 2019). This heterogeneity could lead to uncertainty and confusion.

Respiratory doctor: You've got two diseases…You've got definitely emphysema…and you know your lung function will never get to 100%…But there is a reversible element and a steroid responsive element here which, if you want to label it asthma… I do not…whatever, it's just a word…

Patient: It's not COPD is it? [Laughs].

(OBS‐PA‐013)

Even after formal diagnosis, many participants described an initial lack of understanding of the term ‘COPD’, its meaning and significance. Lack of discussion of disease trajectory or prognosis at diagnosis could consequent lack of understanding about its potentially life‐limiting implications:

Patient I had heard the term [COPD]. It wasn't something I had any particular knowledge of… The first indications were a GP saying, ‘Well, you know your respiratory really ought to be a bit better than it is’. That was the diagnosis…I felt very strongly later that what I needed was a hard, sharp shock ‘You've got the onset of something really serious here, and if you do not take it…seriously *now* this is probably going to be what kills you’, and that just was not said, not at all. (INTS‐PA‐007)

Uncertainty about diagnosis was often bound up with the absence of proposals for treatment. People in the early stages of COPD frequently did not question this. They saw their symptoms as natural sequelae of smoking, to be endured and accepted, rather than identified as an illness and treated as a priority. Indeed, many participants described being explicitly told by clinicians in primary care there were *no* available treatment options for COPD:

Patient: …I said, ‘Is there any more you can do?’ [Nurse] said, ‘Well, not really.’ She said, ‘What do you want me to do?’ I said, ‘Well, help me breathe.’ ….even the doctors…’Oh, well, you have got COPD, you just get on with it’, you know?’ (INTS‐PA‐004)

Treatment appeared to become more of a priority when pathophysiological deterioration meant physical function was more profoundly affected. Then, participants reported an, often challenging, process of re‐engagement with healthcare, sometimes having to negotiate and renegotiate barriers to access specialist healthcare services. One participant who had both lung cancer *and* COPD, was only able to access specialist respiratory healthcare for COPD after undergoing treatment for lung cancer:

Patient I did not have any support [for the COPD] until, really, I had the [lung] cancer… It [healthcare support] all stemmed from that… (INTS‐PA‐004)

Once access to specialist respiratory healthcare had been negotiated, specialist clinicians made a range of treatment options available. Awareness treatment options were available could bring hope to participants but, through engaging with those options, participants began to appreciate the seriousness of their condition, understanding it was incurable, progressive and ultimately lethal. Some participants found it dispiriting treatment was not going to result in a cure. This was compounded by the gradient of disability associated with illness trajectories of long duration. Moreover, unlike people with lung cancer, people with COPD and their caregivers had to balance demands of care against the workload of daily life.

After diagnosis, people with lung cancer found specialist healthcare became almost immediately available. In contrast, participants with COPD had to work hard to access healthcare, often having to exercise considerable effort in securing professional assistance and cooperation. Once participants had obtained access to specialists, their capacity to manage their illness was usually enhanced. Negotiating with specialists and generalists seemed to be an important feature of experiences of people with COPD. Participants and family members often had to follow up recommendations made by specialists and navigate complications arising from communication deficits between primary and specialist care:

Patient …I did have a problem…the [specialist respiratory practitioners] … said I was to take two antibiotics straightaway…and then one in the morning and one at night…The [GP] insisted, ‘No, you take two straightaway and then one a day’, and he would only prescribe me the—I think was nine, and not the 14…I needed….I just used some out of the rescue pack, and I always kept a pack in front of him, if you know what I mean? A bit naughty, I know. (INTS‐PA‐004)

Participants with lung cancer did not seem to experience these complications, and entered into a well worked out pathway. Their engagement with treatment was highly medicalised and generally hospital‐based. Clinicians negotiated and agreed treatment options, along with division of treatment work amongst patients and family members, who often shared tasks. This seemed to be an important focus for outpatient appointments. Alternatively, clinicians and patients could be co‐present, but be required to perform different tasks, for example chemotherapy required specialist knowledge of clinicians to prescribe and administer while patients and caregivers needed little expertise, but often had to do a great deal of work to prepare and attend for treatment. Clinicians delegated few treatment tasks for participants with lung cancer and their family members to carry out at home.

Participants with COPD, however, had very different experiences. Clinicians delegated most treatment tasks to them and their family members to undertake at home. These primarily involved numerous and extensive changes to health behaviours. Clinicians expected participants to exercise, manage their weight and stop smoking. While these health behaviour changes might be supported by healthcare resources (e.g. pulmonary rehabilitation), participants were expected to continue independently at home. These changes to health behaviours were less obviously treatment tasks compared to others, for example managing medications, and were often not recognised by participants and caregivers as treatment, and so not given priority.

Clinicians also expected participants with COPD to develop more sophisticated monitoring and help‐seeking health behaviours at home than those expected of participants with lung cancer. Participants with lung cancer were generally given simple pathophysiological parameters to measure and, invariably, a named nurse and dedicated phone number to call. In contrast, participants with COPD were expected to accumulate a detailed knowledge of ‘normal’ symptoms for them (e.g. sputum colour) and monitor these daily to identify deterioration signalling an exacerbation. Participants' family members shared this work of monitoring and help‐seeking. If deterioration occurred, participants and family members were obliged to make *clinical* judgements about next steps—for example starting medication at home. In outpatient consultations, clinicians frequently evaluated participants against their performance of these delegated tasks. Participants with COPD reported being ‘told off’ by clinicians (and family members) in situations where they had not performed delegated tasks adequately:

Patient …when my breathing gets worse and I start coughing up more coloured sputum, and when my oxygen readings with me finger thing are not very good, it's then I should do something about it. I have to hold my hand up and say I do not…I delay it sometimes longer than I should…That's me own fault, that's nobody else's fault at all, and that's when I get told off! …So, I get told off by the wife, I get told off by the daughter, I get told off by the son. I get told off by the GP. When the ambulance guys come here, they tell me off. When I go into hospital they tell me off, and fair play to them, I do not mind. (INTS‐PA‐001)

### 3.2 Capacity and the relational contexts of patient and caregiver work

Participants with lung cancer were almost exclusively supported by specialist clinicians (doctors and nurses) whom they saw repeatedly, often having appointments every 3–4 weeks, and, consequently, with whom they were able to build a rapport. Most emphasised the importance of support from empathetic, specialist clinicians to whom they were known, and in whom they had confidence. Participants appeared rarely to have contact with their GP and, when they did, were often anxious about perceived lack of familiarity with their disease and its treatment. In addition to regular face‐to‐face appointments with specialist clinicians, participants with lung cancer and their informal caregivers were able to contact a named nurse specialist:

Patient I was assigned a nurse contact… She had a phone number and email, that I could get in touch with her if I needed to, and I did…because I think it just gets really complicated…she was there for the practical side of things, but actually that's what I needed, really…because…you do feel… like a boat tossed in a storm…it's nice just to have someone to check where am I meant to go and have an appointment at 10 a.m. (INTS‐PA‐017)

Although participants and family members gave priority to lung cancer and its treatments, specialist clinicians encouraged participants to pursue other priorities, working hard to provide participants with a flexible and responsive treatment experience, organising or rearranging treatments around competing status passages such as employment, and encouraging holidays:

Lung cancer doctor If you can just forget you have got cancer and get on with your life… I spend a huge amount of my time talking about bloody travel insurance. They cannot get travel insurance to go on holiday…why keep someone alive for an extra ten years if they cannot do anything nice… What's the point in them having this treatment if they sit at home…I really feel strongly about that. My job is to try and let them get a few more years of good quality life. (INTS‐CL‐004)

In contrast, because of COPD's duration and chronicity, participants with COPD saw their specialist clinicians less frequently. They were often obliged to update clinicians about changes to their medication, co‐morbidities and even their disease status that had arisen in the interim. Some might not see the same specialist clinicians and saw this lack of continuity as challenging. Like participants with lung cancer, participants with COPD valued relational continuity with specialist clinicians to whom they were known. Indeed, participants might chose to assume additional work to maintain relational continuity with specialist clinicians. Sometimes they declined medical appointments closer to home, trading this off against extra work they would need to undertake in order to re‐establish relationships with new clinicians. Although individual clinicians tried to provide as flexible a service as possible to their patients, organisational inflexibility of health services made it difficult for clinicians to tailor service provision to individual need. This lack of flexibility made the work of undertaking treatment more challenging for participants with COPD.

The support participants with lung cancer received from family and friends enhanced their capacity to meet treatment demands for their illness. Many participants reported how family members temporarily suspended other activities in order to support them:

Patient My husband and my son, bless them, had to make sure I got there [to treatment]…and got home every day…My husband changed shifts so…he was working nights instead of days so that he could take me (INTS‐PA‐019).

This support was emotionally and practically important in helping participants cope with disease progression and complex treatments. Participants with lung cancer observed were usually accompanied by a family member or friend to clinic. Partners tended to play a significant role in outpatient consultations, often doing much of the work of symptom reporting. They often used the pronoun ‘we’ when responding to clinicians' questions, emphasising the sense of collective participation in treatment. Adult offspring also took an active role, taking notes of discussions and asking questions.

Participants with COPD also found the support they received from family and friends enhanced their capacity to manage their treatment workload. But neither they, nor their family members were able to suspend other demands on their time and energy. People with COPD observed were less often accompanied by a friend or family member to their clinic appointments. Where consultations also involved family members, family could contradict participants' testimony, reporting patients' inabilities to meet the negotiated delegated workload agreed with clinicians.

Participants with COPD reported family members could be required to assume participants' share of domestic workload as pathophysiological deterioration increased. Family members could also be obliged to support participants with complex treatment tasks at home: managing health technologies (such as oxygen), monitoring for signs of deterioration, advising on taking medication, and help‐seeking in emergencies. Demands of multiple workloads could be, at times, very hard work for family members and could accumulate over illness trajectories.

### 3.3 Stigma and isolation

Illness identities in both lung cancer and COPD were bound up with social and psychological mechanisms that acted to separate patients from their social networks. This had significant effects on their capacity to participate in their life worlds. Participants with COPD reported how the capacity available to manage their illness and its treatments could be diminished by diagnosis, through the stigma of guilt and shame of having a ‘self‐inflicted’ disease:

Patient …I was then told I was suffering from COPD. It's smoking related…I remember being quite shocked, and ashamed to a degree. I think this is very much an element of people with COPD that have been smokers—self‐blame, you know, and not expecting any sympathy, really… there's an element of: Well, it serves you right. You smoke….I feel responsible… (INTS‐PA‐005)

Participants also reported experiencing stigmatising attitudes from clinicians and general public. Participants with lung cancer seemed less likely to report these kinds of experiences, although they also expressed feelings of self‐blame and shame. Stigma was not the only separating mechanism shaping patients' social worlds. For some participants with COPD, their social horizons drew in and their social networks contracted as they attempted to avoid possible exacerbation triggers and being marked by visible effects of disease progression and pathophysiological deterioration. Participants with COPD described reluctance to holiday as this highlighted physical limitations which familiar environments disguised. Some would not leave their house because of disabling symptoms. Social isolation affected both participants and caregivers, and diminished capacity to participate in their own care. But not just that: as stigma, social isolation, and logistical difficulties of treatment workloads all acted to limit their social horizons, so too was their ability to access and mobilise capacity diminished.

## 4 DISCUSSION

In this article, we have compared aspects of lived experiences of treatment burden amongst patients and caregivers living with lung cancer or COPD. These are very different diseases and, as we have shown, treatment burden is shaped by illness identity and social context, as well as treatment workload and capacity. These findings have contributed both to the development of our knowledge of treatment burden in respiratory disease and our conceptual understanding of treatment burden. Our previous work demonstrated a paucity of primary studies examining treatment burden in respiratory disease, both COPD and cancer. One qualitative study of burden of treatment in COPD has been undertaken in Australia (Harb & Dobler, 2017) while another retrospective cohort study using a Medicare‐linked database to quantify treatment burden in lung cancer has been undertaken in the USA (Presley et al., 2017). Findings from Harb and colleagues echoed the findings of this study. Participants found the nature of the treatment workload—tasks that involved changing or maintaining health behaviours—particularly challenging. Patients had to rely on, sometimes absent, family members in order to meet the demands of this treatment workload. Presley and colleagues also concluded that patients with lung cancer experienced substantial treatment burden. They defined treatment burden in terms of *volume*: days of contact with health system, number of physicians involved in care, number of medications prescribed. As in this study, the authors found that lung cancer patients spent considerable time interacting with the healthcare system (1 in 3 days during the first 60 days of treatment). In previous literature, treatment burden has been predominantly characterised in relation to workload. So, treatment burden was defined by Eton and colleagues in 2012 as ‘the workload of health and its impact on functioning and well‐being’ (Eton et al., 2012) (p.40). This definition, with its emphasis on treatment burden as the *workload* of healthcare, has persisted in the literature Boehmer et al., 2016; Eton et al., 2012, 2015, 2017; Gallacher et al., 2011, 2013, 2018; Harb & Dobler, 2017; Lorenz et al., 2019; Ørtenblad et al., 2018; Sav et al., 2015, 2017). Importantly, our findings move beyond a simple equation of treatment workload with treatment burden.

### 4.1 Biographical disruption and biographical erosion

The relational consequences of the diagnostic moment are profoundly different. Diagnosis of lung cancer involves a sudden but supported confrontation with an intractable pathology, an existential threat. Bury's influential paper characterises the experience of being diagnosed with illness as a ‘biographical disruption’ where the individual must fundamentally rethink their ‘biography and self‐concept’ (Bury, 1982, p.169). In lung cancer, experience of diagnosis is profoundly disruptive. Thereafter, cognitive and material practices of care are densely concentrated in time and space, and patient work is focused on participating in well worked out care pathways, guided by specialists.

In contrast, patients and caregivers become aware of COPD much more slowly, sometimes without even a formal moment of diagnosis. Unlike the biographically disruptive experience of lung cancer, there is a long period of poorly informed and ambiguous illness experience: biographical *erosion* rather than disruption. Alongside this, cognitive and material practices of care are loosely distributed across a steadily increasing gradient of disability, with infrequent contact with specialist clinicians. Participation in such care demanded Sisyphean labours from people with COPD and their caregivers, and this is contextualised by gradual reconfiguration of their social relations and horizons.

The implication of these very different experiences of biographical disruption and erosion is that relational consequences of diagnosis do not settle illness identity once and for all, but are formative. In this study, patients and caregivers experienced irreversible transitions from one status passage to another and found normative expectations of performance and participation were intimately linked to illness identity and legitimacy of help‐seeking behaviour. What followed from this was, however, negotiable in different ways. This negotiability seems to be derived not from how patients and caregivers manage workload, but rather how they first identified and then mobilised capacity.

### 4.2 Patient and caregiver capacity

Once diagnosed, participants with lung cancer found healthcare almost immediately available; they were not obliged to mobilise capacity. A well‐defined and highly structured treatment pathway was available to them, in addition to practical and emotional support from a team of specialists with whom they were able to develop relational capacity. Specialist clinicians appeared to have discretion to allocate healthcare resources considering patient priorities other than treatment, thus providing a flexible and responsive treatment experience, tailored to individual needs. Practical and emotional support from family and friends was also readily available, with close family members often being able to suspend temporarily demands of daily life to support patients in managing treatment workloads. Indeed, our data suggest families assumed *collective* illness identities, allowing for collective action and bolstering patients' structural resilience (their potential to deal with adversity) (May et al., 2014). Participants did not report being held culpable for their disease, instead capacity was directed towards managing an intractable pathology (lung cancer).

In contrast, participants with COPD were obliged to exercise considerable social skill in order first to identify, and then mobilise, capacity to access healthcare. Participants had to engage and re‐engage with healthcare providers in order to be given and, subsequently, understand their diagnosis and secure access to different treatment options. Once treatment options had been identified, participants with COPD had to work hard to access a fragmented and confusing treatment pathway which they themselves had to co‐ordinate between primary and secondary care. Where relational continuity was established, participants valued support from clinicians in both primary and secondary care. However, clinicians appeared to have less discretion than those in cancer services to allocate healthcare resources, meaning treatment experience could appear inflexible. Practical and emotional support from family and friends were highly valued but family members' capacity to support patients could itself be diminished by multiple workloads. Rather than assuming a collective illness identity as in lung cancer, our data suggest a clear separation in identity between patient and family member with respect to COPD. Participants reported feeling ‘told off’ by both clinicians and family members for failure to perform against agreed treatment tasks which might diminish their structural resilience. Structural resilience had already been diminished by internalised stigma, where participants blamed themselves for their ‘self‐inflicted’ smoking‐related disease. Participants felt culpable for their disease and were themselves deemed intractable patients by clinicians and family members when they were unable to meet demands of treatment workloads.

### 4.3 Priority given to treatment workload

For participants with lung cancer, the threat of death and the hope treatment might be life‐prolonging or even be curative meant participants were allowed and, indeed, *expected* to adopt a more traditional sick role. Thus, they were temporarily exempted from demands of other status passages in order to prioritise treatment of their illness (Parsons, 1964). Despite a heavy treatment workload with potentially debilitating pathophysiological side effects, participants could be reluctant to stop treatment as this could be tantamount to accepting death. Participants did not appear to view heavy treatment workloads as burdensome but rather as providing hope. Conversely, participants with COPD initially had little or no understanding of the meaning of their disease and its implications. Unclear, uncertain and often prolonged illness trajectories meant participants were obliged to balance demands of treatment workloads with those of daily life. When participants with COPD did gain knowledge about the progressive, potentially lethal nature of their disease and its trajectory, its treatments and their limited curative value, this could take away hope and, consequently enthusiasm for undertaking the demands of treatment workloads.

### 4.4 Strengths and limitations

This study is limited by its cross‐sectional rather than longitudinal design. Longitudinal designs may be particularly well suited to understanding the evolving and dynamic nature of treatment burden. An additional limitation may be the fact that it was not clear what stage of disease participants interviewed were as the research team did not have access to their clinical records. The key strength of the study is the abductive approach taken to study design, data collection and analysis (Tavory & Timmermans, 2014). This has meant that we have built iteratively and recursively on systematic reviews to enable robust, empirically and theoretically informed characterisation of constructs of workload and capacity in lung cancer and COPD which has extended and enhanced our understanding of treatment burden.

## 5 IMPLICATIONS FOR POLICY AND PRACTICE

This has important implications for healthcare practice and policy. We began by setting out the sociological background to the work of patient‐hood. This understanding of treatment as work is not always appreciated in healthcare settings, particularly in relation to delegated tasks. Indeed, much of this workload may be invisible to healthcare professionals who may not realise its impact on patients and informal caregivers, particularly over time (Dobler et al., 2018). Healthcare professionals should recognise this delegated workload might be experienced by patients living with chronic illness as hard, relentless, lifelong work, and as potentially burdensome as the obvious workload of treatment for cancer. The design of health care systems and organisations is an important exacerbating factor in treatment burden (Gallacher et al., 2018). In this paper, participants described how fragmented, poorly coordinated services, organisations and healthcare professionals which operated independently of one another added to workload and reduced capacity. Since this study was undertaken, the COVID‐19 pandemic has dramatically affected the way in which healthcare is coordinated and delivered to patients, both respiratory and other, for example the rapid rise in care being delivered virtually through telemedicine (Pierucci et al., 2021). This increased remote delivery of healthcare underscores the importance of the consideration of treatment burden in clinical practice. It is vital that healthcare professionals understand the treatment workload that they are delegating to patients and their informal caregivers and the capacity that patients and their informal caregivers have to meet the demands of this workload. Moreover, the pandemic has demonstrated the importance of not only acute care for patients in hospitals but, as an increasing body of evidence is demonstrating, complex, integrated care over time for patients experiencing Long Covid (Maxwell & Radford, 2021). This resonates with the findings of this study. We argue, therefore, for a fundamental shift in healthcare design, so healthcare systems not only provide for patients with acute conditions who need episodic, short‐term care but also supply life‐long, holistic care required for those with life‐limiting conditions.

## 6 CONCLUSION

By comparing and contrasting constructs of illness identity, capacity and workload in lung cancer and COPD, we have deepened our conceptual understanding of treatment burden. This study has shown that treatment burden is not, as some suggest, simply the work patients have to do to meet treatment workload demands. Our comparative analysis finds diagnosis and illness identity affect the priority that patients, family members, clinicians and society itself give to meeting the demands of treatment workloads. Thus, treatment workload in lung cancer may bring hope rather than burden. In COPD, treatment workloads must be balanced with demands of daily life and may, therefore, accumulate over the uncertain, but often long, illness trajectory to burden patients and their family members. Diagnosis and illness identity may also affect the nature of treatment workloads, so in chronic non‐malignant conditions, tasks may be delegated by clinicians to patients to manage independently at home over lifespans. In lung cancer (and possibly other cancers), temporally limited tasks are more likely to be undertaken by clinicians and patients in hospital together. Diagnosis and illness identity may also affect patients' capacity to meet the demands of treatment workloads and, crucially, the ability to mobilise this capacity and the structural resilience required subsequently to sustain it.

## FUNDING INFORMATION

Kate Lippiett was supported financially by the Health Foundation. Kate Lippiett also received support from the NIHR Applied Research Collaboration ARC Wessex and Health Education England South East and was funded by an NIHR ARC Wessex and Health Education England South East Internship. Carl May's contribution to this article was supported by NIHR North Thames ARC. Alison Richardson's contribution to this article was supported by NIHR ARC Wessex. Professor Richardson is a National Institute for Health Research (NIHR) Senior Investigator. The views expressed in this article are those of the author(s) and not necessarily those of the NHS, the NIHR, the Department of Health, HEE South East or the Health Foundation.

## CONFLICT OF INTEREST

Kate Lippiett reports personal fees from Glaxo Smith Kline, grants and personal fees from Boehringer Ingelheim, personal fees from Teva, outside the submitted work. Carl May and Alison Richardson report no conflicts of interest.

## Supporting information


Appendix S1

Appendix S2

## Data Availability

Much of the data that support this article is available in the supplementary material in the appendices. Further data are not publicly available due to ethical restrictions.
