# Supplementary material for: How do illness identity, patient workload and agentic capacity interact to shape patient and caregiver experience? Comparative analysis of lung cancer and chronic obstructive pulmonary disease
Source: Health Soc Care Community. 2022 May 28;30(6):e4545–55. doi: 10.1111/hsc.13858 (PMC10084268; doi:10.1111/hsc.13858)
Supplement: Supplementary file 1 — Appendix S1 [file HSC-30-e4545-s001.docx]

Appendix A: preliminary coding framework

| **Pathway and capacity** | |
| --- | --- |
| 1.1 Pathophysiological status | 1. Involuntary diminution of physical and cognitive function brought about by the natural progression of the disease. This includes the psychological impact of symptom exacerbation, disease progression, and aggressive treatment on the patient. |
|  | 1. The impact of involuntary diminution of physical and cognitive function brought about by symptom exacerbation, disease progression, and aggressive treatment on the caregiver. |
| 1.2 Status passage | Processes taking place over time and across settings in which the patient or caregivers’ identity and status are reformulated through the effects of institutionally defined and sanctioned interactions, relationships, and practices. |
| 1.3 Personal capacity | Personal resources (which may be affective, cognitive, informational, material, physical and relational) that are available to be mobilised by patients/caregivers. |
| 1.4 Distributed capacity | Resources (which may be affective, cognitive, informational, material, physical and relational) that are available to be mobilised by members of patients/caregivers’ wider social networks` |
| 1.5 Workload | Goal-oriented affective, cognitive, informational, material, and relational tasks that are assigned to patients/caregivers |
| 1.6 Interactional quality | Experienced interaction quality with healthcare workers |
| 1.7 Structurally induced non-adherence | Non-adherence to treatment due to structural factors such as having insufficient capacity to adhere to treatment regimes |
| 1.8 Volitionally induced non-adherence | Non-adherence to treatment due to personal choice |
| **Context** | |
| 2.1 Action environment | Physical or virtual transaction spaces in which patients/caregivers interact with each other, mobilise capacity, and perform tasks. |
| 2.2 Structural advantage | Structural advantage: social structural factors that shape personal and distributed capacity, including structural inequalities (socio-economic status, education, gender, sexuality, ethnicity, age); access and location (proximity to health services, housing class, quality and availability of transport); and health system (availability of service, cost of service, quality of service). |
| **Power and control** | |
| 3.1 Experienced control | Patients/caregivers’ ability to achieve goals within an action environment or across a status passage by influencing the beliefs and actions of relevant others. |
| 3.2 Accountability | The ability to mobilise capacity (affective, cognitive, informational, material, physical and relational resources available to patients or care-givers) in relation to expected beliefs, behaviours, responsibilities and actions. |
| 3.3 Negotiated obligations | Negotiated agreement about of future actions and the degree of accountability (expected beliefs, behaviours, responsibilities and actions). |
| 3.4 Cognitive authority | Patients/caregivers’ ability to define and determine goals, workload, resource mobilisation, (and to resist others’ attributions of expectations and responsibilities) in any given action environment. |
| **Burden** | |
| 4.1 Mapping emergence | Adaptive and reflexive behaviours and practices through which patients/caregivers apprehend changing self-identity and work with changing attributions about their identity and status, and about the nature of self-identified and institutionally sanctioned goals, workload and accountability that stem from these. |
| 4.2 Resource identification | Adaptive and reflexive behaviours and practices through which patients/caregivers define and determine their wants and needs in relation to their personal and distributed capacity (affective, cognitive, informational, material, physical and relational) to meet self-identified and institutionally sanctioned goals, workload and accountability. |
| 4.3 Resource mobilisation | Adaptive and reflexive behaviours and practices through which patients/caregivers operationalise elements of personal and distributed capacity (affective, cognitive, informational, material, physical and relational) to meet self-identified and institutionally sanctioned goals, workload and accountability. |
| 4.4 Relational monitoring | Adaptive and reflexive behaviours and practices through which patients/caregivers make sense of interactions and relationships between self-identified wants and needs, capacity and workload, cognitive authority and experienced control. |
