# Supplementary material for: How do illness identity, patient workload and agentic capacity interact to shape patient and caregiver experience? Comparative analysis of lung cancer and chronic obstructive pulmonary disease
Source: Health Soc Care Community. 2022 May 28;30(6):e4545–55. doi: 10.1111/hsc.13858 (PMC10084268; doi:10.1111/hsc.13858)
Supplement: Supplementary file 2 — Appendix S2 [file HSC-30-e4545-s002.docx]

Appendix B: Taxonomy of treatment burden with associated exemplar quotes

| **LUNG CANCER** | **Exemplar quote** | **COPD** | **Exemplar quote** |
| --- | --- | --- | --- |
| Diagnosis as shock | Patient: I don't know, because I think at first I didn't want to believe, and I couldn't believe that I'd had this [diagnosis of lung cancer], and I was *absolutely*, and I'm going to be honest, I was terrified. And I don’t think…it's almost like I was in a different world, to be quite honest. Even though people were stood in front of me, or sat in front of me talking, I just was *unable* to take anything in... I felt as though I was in a different - I don't know, even though we were all in the same room, it was almost like were in parallel worlds (INTS-PA-010) | Diagnosis imperceptible | Patient: Actually I don't really [know who gave me the diagnosis of COPD] - oh, I think the nurse suspected it to start with.  Researcher: At the GP surgery?  Patient: Yes, because I'd always been struggling…she thought this a while back but it was only when I went with this chest infection that they got really worried…I think it was there [at the hospital admission following the chest infection] that it was more brought up as COPD because of the breathing aspect (INTS-PA-013) |
| Obvious illness identity with socio-cultural resonance (therefore understood by patient/informal caregiver/HCP) | Wife of patient: …That was a shock that time then, being told he had cancer.  Patient: …I had an appointment with some, I think that was somebody else down there and I walked into the room and I remember the specialist nurse. I walked into the room with him and the doctor and she said, 'We can't cure your cancer,' she said, 'But we can treat it,' and so we never had no inclination that I had cancer at that point, of course my daughter was with me and we were all upset, it was a bit of a shock…It was a bit of a shock to say it…  (INTS-PA-018) | Unclear illness identity, without socio-cultural resonance (therefore poorly understood by patient/informal caregiver/HCP) | Patient: Right, as I explained to somebody the other day, everybody has heard of the big C, cancer, if you say cancer everybody, 'Oh, terrible,' yes… if somebody says, 'Well, he's got cancer,' they all go, 'Oh, well that's fine, ah yes, that makes sense.' Maybe COPD and pulmonary disease isn't, things like that, need to be, I was going to say advertised but that's not… It needs to be made, people need to be made more aware of it. (INTS-PA-012)  Wife of patient: Well, I have never really understood, for a long time, what COPD meant. I knew it was chronic obstructive pulmonary disease but that doesn’t really tell you very much” (INTS-PA-009). |
| Short disease trajectory (clear to patient and informal caregiver) | Patient: I was told [of my diagnosis of lung cancer] …Of course, it hit me [sound of clicking fingers] and it was all going round…She [the lung cancer nurse specialist] came - the doctor told me and she stayed with me, and I said, 'Well, is it operable?', 'No', 'Is it curable?', 'No', 'Well, how long have I got?', 'Oh, it could be days, it could be weeks', and that really…and of course, well, they say your life flashes before you. Everything, I thought my God, I'm not going to get out of here, I'm not going to see my sons and that carry on, and everything just whirled. That night, I didn't sleep much at all. (INTS-PA-020) | Long and uncertain disease trajectory (unclear to patient and informal caregiver) | Patient: I know the disease I've got is incurable, I know it's progressive, so if someone says to me, 'You've got a year to live,' fine, quite happy with that. I'd whoop it up a bit. That's the sort of information I want to know. I think the problem is, why they don't do that is because they can't give you a treatment to dangle in front of you to say, 'Well, if you do this, it might go from a year to three years.' I think that's where the problem is… (INTS-PA-001) |
|  |  | Diagnostic ambiguity | Wife of patient: The consultant walked into the room and said to him “You do realise you’ve got severe emphysema, don’t you?” And although I was taken aback, I was pleased because at last I had something that I could understand. And explain to others, so that when they said to me, “What’s wrong with [name of patient]” I said, I can say “He has severe emphysema”, and most lay people do know that term, so it makes them more sympathetic and understanding than COPD. (INTS-PA-009) |
| Demands of treatment workload as overriding life priority (for both patient and informal caregiver) | Patient: my husband and my son, bless them, had to make sure I got there [to treatment] every day and got home every day…My husband changed shifts so that he was working nights instead of days so that he could take me during the day (INTS-PA-019) | Demands of treatment workload balanced with domestic/professional/sentimental demands of everyday life (for both patient and informal caregiver) | Patient:…when I was talking to [name of respiratory physician] recently, had I been on [pulmonary rehabilitation] rehab for my condition, yes…when I first did it it wasn't so bad, it was done once a week every week, I think it was half past five at [name of local community hospital]. What I actually got my then boss, my then director to agree was I would slope off at four o'clock, my Mrs would pick me up, I'd be at [name of local community hospital] by five o'clock. One day a week for the, I think it was an eight or ten-week period, that's what I would do. Now when I looked into it next and when I've looked into it again recently, yes, they do it, it's something like two hours a day [twice a week] and you do that for five weeks…so that would mean I'd have to say to my bosses, 'Do you mind if I take ten days off in the next five weeks at your expense or full pay?' or I'd have to book them as holiday, and as I don't have ten days holiday, guess what, I will not be going on this rehab thing, you know what I mean? It's something that doesn't take an Einstein, I couldn't do it even if I wanted to. Now who in their right mind decided, 'Do you know what, if somebody needs to go on this two hours a day, twice a week for five weeks'? (INTS-PA-012) |
| Practical demands of treatment workload as a relief from the existential threat of cancer | SYSTEMATIC REVIEW ONLY | Practical demands of treatment workload as hard work | Patient: Was this in [local district general hospital]? Last time I was up in [local district general hospital] yeah and he sent me home while I was still ill. But er I went to that um you know two a week exercise thing.  Specialist respiratory nurse: Oh right? At [name of pulmonary rehabilitation centre]  Pt: Yeah and I went [two days of the week classes held]  Specialist respiratory nurse: Yes, that was with us. Yes, OK  Patient: And I wasn’t well in any of them.  Specialist respiratory nurse: No, you weren’t.  Patient: No [laughs]. And I was gasping…  Specialist respiratory nurse: But you still came didn’t you?  Patient: Yes.  Specialist respiratory nurse: You did very well.  Patient: I missed three. One was a hospital visit and two was I just didn’t fancy the idea of you putting me through the torture  (OBS-PA-002) |
| Treatment as hope | Patient:...I was taken aback a bit about - he [specialist oncology doctor] give me some treatment which made me rough, but I didn't mind. I didn't want it, but put up with it because that's the way it is. He said, 'We'll stop that.' I said, 'Well, you know,' and he said, 'Well, I'm not sure that it's doing any good anyway.' Yes, but, I just feel that I should have carried on with that treatment a bit more because there was more pieces to it. I never got it. He changed it to something else. So, I don't know. My biggest fear is that it comes to the end, and he says, 'I've done all that I can,' and it hasn't done a great deal of good, do you know what I mean? (INTS-PA-016) | Institutionalised care as respite from unrelenting demands of self-management | Patient: …we were in the garden and obviously it was August, it was summer, and I was out there sawing a piece of wood and the following couple of days my shoulders had started to ache and I put it down to the fact, 'Oh I've just done something sawing a piece of wood,' strange as that seems. Obviously, that was masking what was actually happening here and it suddenly caught me out, I suddenly realised it wasn't the shoulder at all, it was my lungs and I got caught. Obviously, I was admitted. I felt, even though I was being admitted, I actually felt okay, you know, like, 'Oh, well I've got to go in. They're going to fix me.' Obviously, they said it was a little bit worse than that and it was lucky that I went in when I did go in because otherwise it could've become even a bigger problem (INTS-PA-002) |
| Sense of ‘limbo’ once treatment completed | SYSTEMATIC REVIEW ONLY |  |  |
| Reluctance to stop treatment despite debilitating pathophysiological side effects | Specialist oncology doctor: So obviously a treatment like this, it’s a new treatment and it’s different to what you had before. As I’ve told you, I wouldn’t anticipate it being any more difficult than anything we’ve done previously but erm you know I appreciate you’re feeling slightly apprehensive and the bottom line with these sorts of treatments is you only know how you are going to get on with it once you’ve tried it  Patient: Yes, that’s what I said to my granddaughter  Specialist oncology doctor: And I suppose the reality is that, you know, in the last few months we’ve been trying to find something to do  Patient: Yeah, that’s right  Specialist oncology doctor: because we’d run out of options and then this was a new option that’s been made available to us.  Patient: That’s it  (OBS-PA-029) |  |  |
| Treatment for family rather than for patient | Patient: They said it was lung cancer and of course I never wanted to, I always said I wouldn't take chemo but when I told my daughters they, 'How could you say that? What about us? What about your grandchildren?' I said, 'Well, I'll see. (INTS-PA-011) |  |  |
| Lack of options: treatment or death | Patient: Well, there wasn't anywhere else to go. I could have said no to [name of consultant giving radiotherapy], when he originally suggested it, and said, 'You have to come to [name of local teaching hospital] for it'. I thought, oh, no. I knew if I didn't have it, I'm my own worst enemy. When something like that is offered to you, there's a reason, it's an expensive procedure, they don't give it to you for nothing.  (INTS-PA-019) | Lack of treatment options (lack of information or feeling that ‘nothing can be done’ from HCPs) | Patient: I was sent to have this x-ray and they diagnosed emphysema. Following that, for many years, nothing really happened. I just carried on as if I wasn’t told anything, it didn’t make any difference to me. I just carried on working, and so on and so on. (INTS-PA-001) |
| Decision to cede control over choice of treatment options to trusted HCPs | Specialist oncology doctor: So if you went up to full dose, you’d have enough for 14 days but knowing you, you’d probably want to increment it I would have thought?  Patient: Well I was going to ask your advice. I thought I was going to have to go up to the top and give it a blast because that’s what is necessary but I don’t think that my system can cope with it  Specialist oncology doctor: Oh, I don’t know. You never know do you? Why don’t you go up to 3 and 3? Or three in the morning, two at night for a couple of days? Then 3 and 3, then 4 and 3, then 4 and 4?  Patient: So how would you like me to…?  Specialist oncology doctor: However you would like to do it. As quickly as *you* think you could do it  Patient: I’m not good at decisions so you need to tell me.  Specialist oncology doctor: [sighs, laughs]  Patient: If you give a suggestion and if I can’t do it, I’ll…  Specialist oncology doctor: Every other day, go up another dose  Patient: And you want me to go up to the top?  Specialist oncology doctor: [short pause] Yeah, I think you need to.  (OBS-PA-033) |  |  |
| Immediacy of availability of specialist healthcare | Patient: I have lung cancer. So I saw [name of respiratory physician], and she arranged for me to see [name of thoracic surgeon] at [name of local teaching hospital], who was a surgeon, and everything seemed to move very, very fast….once they'd discovered what it was…So I said to [respiratory physician] at the time, knowing what the NHS was like with operations, et cetera, 'How long will it be before I see him?' and she said, 'If you haven't seen him within two weeks of today's date, ring my secretary', which surprised me. Within ten days I was talking to him (INTS-PA-014) | Work (for patients and informal caregivers) of accessing healthcare | Patient: The GP…recommended that I did [pulmonary rehabilitation], and nothing happened, and this did happen occasionally at the surgery…but I was then turned down, several times, because of the state of my chest and I was on medication and so on. Then I finally got in a number of years after it was first mooted…. (INTS-PA-009) |
| Specialist HCPs with specific knowledge of lung cancer | Patient : I think my first time of meeting [name of specialist oncology doctor], and I think I remember saying to him, 'I just would like you to treat me as though it was a member of your own family', and he has done. He shook my hand, and I know he's very passionate about this disease. So I would say that I had that immediately. I just felt that warmth from him. I don't know what it was. I just knew that…I feel very, very comfortable there [in the hospital].  (INTS-PA-010) | Doctors/nurses in primary care who lack specific knowledge of COPD | Patient:…I said, 'Is there any more you can do?' [Practice nurse with responsibility for respiratory patients] said, 'Well, not really.' She said, 'What do you want me to do?' I said, 'Well, help me breathe.' And she said…I said, 'Oh, well, don't worry about it', so that was that….even the doctors, I don't think - you know, kind of, 'Oh, well, you've got COPD, you just get on with it', you know? 'Just take it easy, keep indoors, rest up, take some paracetamol, and have your puffers, and just get on.' (INTS-PA-004) |
| Structured treatment pathway | Specialist oncology doctor: Yeah. How many have you had [cycles of immunotherapy] now? Two or one?  Patient and Informal caregiver: Two  Specialist oncology doctor: Yeah. Good. So, you’re due a scan after four, that’s the plan which will be in about 5 weeks’ time. I’ll just print the form off and we’ll just carry on and we’ll see you back here in 3 weeks  Patient: Lovely  (OBS-PA-028) | Fragmented treatment pathway | Daughter: The other problem that we have is that when you prescribed her the B…whatever it is you prescribed her she was given [other inhaler name] and took that. So for 2 weeks she was taking both. And the locum doctor flagged her up and confused her over the phone and that’s when I got in touch with your thingy. So she’s now taking [inhaler name]. She’s taking 2 in the morning and 2 in the afternoon  Patient: At night  Daughter: Oh sorry, 2 in the morning, 2 at night. Now when she was taking both inhalers she was fine on it and she’s felt fine haven’t you?  Patient: I had no idea that I was taking the wrong inhaler  Specialist respiratory doctor: No, no, no. All you were doing a little bit was doubling up on one of the medications which is…  Patient: That’s what he said  Daughter: Not dangerous  Specialist respiratory doctor: Not dangerous, it’s fine….  Daughter: So she’s back on the [inhaler name] which is what she should be on  Specialist respiratory doctor: She should be on  Daughter: And the [inhaler name].  Specialist respiratory doctor: Yeah  Daughter: All sorted I think. I’ve got a repeat prescription in my purse for the [inhaler name] which [GP name] gave her just so that she would never run out. Now that’s a 50 dose  Specialist respiratory doctor: Yep  Daughter: which obviously if you’re taking 4 puffs a day will only last 12 days isn’t it?  Specialist respiratory doctor: That’s ridiculous. (OBS-PA-021) |
| Specialist treatment workload in secondary care with debilitating pathophysiological side effects | Patient: You have the chemo, and then you don't even know you've had it, and then, say, you have it on a Monday, you're all right, and then by about Thursday, Friday, Saturday you start feeling rough, not well. By Sunday and Monday you feel quite ill. Lifeless, no energy. All I wanted to do was lay on the settee, and I really felt ill. That lasts for about three or four days, four or five, and then - you see, what happens with chemo, as you probably know, it kills all the white corpuscles. The white corpuscles grow back, the chemo that it's killed doesn't. So you've got no white corpuscles, so you feel very, very ill. Your white corpuscles start growing back, so you start coming back up. So by the end of the second week, if you like, you start feeling fine. Then you're all right for a week, and then you go and see them and they do it all over again. That's how it went on. (INTS-PA-014) | Multiple appointments for treatment in primary, secondary care and in the community | Researcher: It seems like your daughters keep you well organised.  Patient: Oh god, yes!  Researcher: Do they keep on top, on track of all your appointments for you, do they?  Patient: Yes, yes. .. [coughs] except we had a cock up today. I've got an appointment with [name of consultant respiratory physician] it was my fault, tomorrow. I thought it was today and we went down there today….I'd written it on the calendar the correct date. I don't know how I got it into my head that it was today, but sometimes I do have quite a few appointments.  Researcher: How do you keep on - do you have a calendar?  Patient: I have a calendar and I also put them in the phone [laughs].  (INTS-PA-003) |
| Limited delegated tasks from HCPs | Specialist oncology doctor: And this is important, erm I know it sounds a bit alarming but essentially any chemotherapy, any treatment we give for cancer carries risk. The biggest risk with this sort of treatment as I said is infection. If someone gets an infection with low blood counts I don’t know if you remember this, we call it neutropenic sepsis so you can get blood poisoning without the blood cells to fight it. And very occasionally people end up very unwell with that and it can become a life threatening problem if it goes untreated. Whenever anyone ends up ill in hospital and we work out what went wrong, nearly always it’s that someone was ill for a few days at home and didn’t get in touch with us. So the thing about chemotherapy-related problems are, if you ignore them and hope that they get better, they tend not to, they tend to get worse, whereas if you contact us then we can normally sort it out. So, I know it’s not nice to talk about these sort of things.  Patient: Well, you’ve got to haven’t you?  Specialist oncology doctor: Well, I think that my responsibility is to make sure, you know, what to do if there is a problem...About 1 in 10 people might get something that we’d need to do something about. And we would expect you to ring up on the phone number you know that you’ve got  Patient: The same as you would do for chemotherapy?  Specialist oncology doctor: Yeah.  (OBS-PA-027) | Significant workload of delegated treatment tasks at home from HCPs | Specialist respiratory nurse: I’m saying if you were to be unwell, you would notice that you would perhaps get more secretions on your chest and you’re quite right, they would change colour if you were to get an infection. So then you would need some antibiotics and steroids.  Patient: I’ve got a kit  Specialist respiratory nurse: You have. I remember, you’ve got a rescue pack. So you would start those yeah?  Patient: Yeah  Specialist respiratory nurse: and let your GP know that you had a chest infection, would you?  Patient: I suppose you would, so that he can renew it.  (OBS-PA-016) |
|  |  | Workload of changing health behaviours at home | Specialist respiratory doctor: Very good. And are you keeping going [with exercise following pulmonary rehabilitation]?  Patient: No.  Specialist respiratory doctor: Why not?  Patient: I dunno [laughs]  Specialist respiratory doctor: What do you mean you don’t know?  Patient: I haven’t done nothing since I finished it.  Specialist respiratory doctor: Well, flipping well…  Wife: Get off your backside and do something. I’m sorry  Patient: I need to go back onto it [PR] then  Specialist respiratory doctor: Well, I can’t do that. Not yet. Not for a year or so but um come on get down the gym, get down the stairs in the hall. You’ve got to do it… we know that this is the most important thing…  Patient: Exercise  Specialist respiratory doctor: Intervention that we can do. Better than any drug. Um and we know that that changes lives. So you’ve changed your life doing the rehab. No question. You’ve changed from here to here. So you’ve got to push on and follow through now.  (OBS-PA-012) |
|  |  | Clinicians performance manage patients against delegated tasks | Specialist respiratory doctor: Ahh. You’ve put on a bit of weight [patient’s name] what’s that about? No, a lot of weight.  Patient: I know! A lot of weight.  Specialist respiratory doctor: What’s?  Patient: Just things have gone wrong.  Specialist respiratory doctor: What’s, what’s going on?  (OBS-PA-001) |
|  |  | Informal caregivers report failure of patients to perform against delegated tasks to clinicians | Specialist respiratory nurse: Are you doing any of those exercises at home?  Patient: Yes, tonnes of them  Wife: No you’re not.  Specialist respiratory nurse: [laughs]  Informal caregiver: [gasps] God’s…  Patient: I get up to the toilet and go back again. That’s walking…  Specialist respiratory nurse: Do you have that book that we gave you with all the exercises in?  Wife: Yeah  Specialist respiratory nurse: Is it gathering cobwebs somewhere?  Wife: Yeah  Patient: No  Wife: Yeah it is. You don’t use it  Patient: *I* don’t, no. Where is it?  Wife: You see. Where is it? He doesn’t even know where it is.  (OBS-PA-016) |
| Generally high quality information provided in written form and from specialist HCPs | Specialist oncology doctor:…this new group of drugs which is called immunotherapy drugs. So these drugs are antibodies, they don’t attack the cancer cells themselves, what they do is they latch onto your body’s immune cells and they basically switch on the immune cells so that *they* attack the cancer. In trials that have been done, we’ve shown that these new immune drugs are better than chemotherapy. And they also seem to have fewer side effects. So it’s a good treatment to be able to have. So the treatment that we are using is a drug called Pembromizulab. It’s an intravenous treatment and it’s done every 21 days so it’s once every 3 weeks. It takes half an hour to give so it’s very quick. So all you have to do is come and see me and then a day or two afterwards you come back and you have a cannula put in and a drip of this treatment put through and then you go home afterwards and I’ll give you some information to read about for that... (OBS-PA-031) | Patients typically poorly informed about condition from diagnosis to death adding to treatment workload | Patient: Just going back to what you were saying about the appointments and stuff like that, I personally don't get a lot from them. I sometimes think it's because they're frightened to tell you the truth.  Researcher: The healthcare professionals are frightened to tell you the truth?  Patient: I do get that impression sometimes…When you go to [name of oxygen assessment location], and you do your six minutes, and they do the saturation and listen to you. They don't actually say to you, 'You're falling apart, you're getting bloody worse,' or they don't say, 'You're improving,' or anything. They ask you about smoking and stuff like this, which is fair enough…  I want to know what's actually going on with my body, for someone to tell me. If it's falling apart I want to know that.  Researcher: What about the doctor at the hospital?  Patient: When I go and see [name of specialist respiratory doctor], we have a chat there, and I get the impression he's reserved on what he says to me. I think he says to me enough to, if you like, satisfy me but he's not giving me the blunt truth. (INTS-PA-001) |
| Lack of information as a deliberate choice on the part of patients – a tactic for maintaining hope in the face of a poor prognosis | Patient: …the surgery was an interesting case in point, because obviously they had gone through what they were going to do and I knew roughly what they were going to do. They had suggested I go away and read some other things, but I found it all a bit intimidating at that stage. So, actually, I didn't, I didn't go on the internet, the website they recommended. I didn't want to go near the internet to be honest because typing the words 'lung cancer' into Google is literally the worst thing you can do [laughing] if it might actually be happening to you…I did feel a bit stupid because I hadn't done as much research as I could have done before my operation because I just didn't really want to know. I felt it might be better to go into it a little bit ignorant, because otherwise it was just too scary. As soon as you start thinking about what's actually going to happen, you get really freaked out. (INTS-PA-017) | Conflicting/contradictory information adds to patient/informal caregiver distress | Specialist respiratory nurse: And how many puffs [of the inhaler]?  Patient: sometimes 4, sometimes maybe 6  Specialist respiratory nurse: What puffs?  Patient: Yeah  Specialist respiratory nurse: In one go?  Patient: Yeah  Specialist respiratory nurse: OK. So that’s not really how we should be using the inhaler. We should only use maybe 2 puffs in one go  Patient: Yeah, I’ve had this….My first doctor told me I was allowed up to 10 puffs  Specialist respiratory nurse: So that’s only if you’re really  Patient: out of puff  Wife: which he gets  Specialist respiratory nurse: So that’s the equivalent of a nebuliser, yeah?  Patient: yeah  Specialist respiratory nurse: which it seems quite extreme perhaps. So generally, general maintenance, if you were out of breath and struggling a little bit, you would just use it 2 times.  Patient: Just a couple of times  Specialist respiratory nurse: I wouldn’t really promote that you use it 8 times in one go because it can have side effects  Patient: Well they tell me it can’t.  Specialist respiratory nurse: OK  Patient: …The nurse at this new doctors. She said up to 8.  Specialist respiratory nurse: OK. Well we’re all singing off different song sheets then aren’t we?  Wife: It’s very confusing for him. He gets very confused.  (OBS-PA-016) |
| Conflicting/contradictory information adds to patient/informal caregiver distress | Patient: I think the main thing is to - because they didn't have all my full results. They were going on the basis of the first result, and I think it would be more beneficial to wait until they'd got the full picture and then tell you what's - because with me they jumped in at the deep end, the worst scenario. Then when it worked out it - well, when I went to see [name of consultant oncologist] the first time, he said, 'Oh, now we've got all the results it's the less aggressive one', whereas they were, I think, going on the aggressive one. It does, if you're not well as well, panic you and pull you right down…You think my God, my world's finished sort of thing, but, yes, I think if they waited until they'd got all the test results and the full picture and give you the correct diagnosis right from the start, then it would - for me, it would work…  (INTS-PA-020) |  |  |
| Family and friends are seen as the main source of support post diagnosis (but fear of being a ‘burden’ on family) | Researcher: You were saying how supportive your family…  Patient: Oh they're marvellous, they are marvellous….I've got step children as well, and they're marvellous as well….the family's lovely, all-important… As I say, the support from the family is very important.  (INTS-PA-015) | Family and friends are seen as the main source of support post diagnosis | Specialist respiratory doctor: Are you still taking that little…I…I…to help you with the swallowing, I gave you a little tablet of antibiotics that sometimes helps swallowing. Has that helped at all? Are you still taking that?  Patient: Yes  Specialist respiratory doctor: Cos again, it’s not on your list.  Patient: The wife’s got them there and the boss feeds them to me.  (OBS-PA-015) |
| Family and friends are able to prioritise supporting the patient through their treatment workload owing to the short disease trajectory and the recognition of the patient’s likely imminent death | Patient: Yes, my son lives three minutes' walk… I've only got to pick the phone up and he'll be there…he's self-employed. I don't like him doing it because he's losing money, isn't he? So, but…I've told him, I'll get transport, I've been offered transport. 'No father,' he said, 'You'll get it all wrong when you've got to go back and all the rest of it.' He said, 'Two heads are better than one. Two heads are better than one,' so, there you go…Well, the thing is there's only my two boys, that's all there is. When my wife was doing it, I went with her every time. He said, 'You've got to have someone with you.' It's no fun playing with chemo, because it's not the best thing in the world, but…he waits with me, yes, yes… but he likes to be there when there's a doctor there giving me information. Yes, he likes to be there see what's going on which is fair enough. (INTS-PA-016) | Family and friends have to balance the demands of the treatment workload with the demands of everyday life owing to the long and uncertain disease trajectory | Wife: …you had a chest infection. We didn't really, we got so used to them, we'd take no notice.  Patient: Yes.  Wife: We were going on holiday with our [name of tour operator] and it was [date] and [name of patient] said to me, 'I don't feel like going but please will you go because I'm always forcing us to cancel things.' We were going to [name of county] and it was Monday to Friday and, anyway, we were having the hall painted and I set off and rang up and said, 'We've arrived.' This was tea time and he said to me, 'Well, you know where I am, don't you?' I said, 'Well, where?' He said, 'I'm in [name of local teaching hospital].'  Researcher: Blimey.  Wife: Because he was in such a bad state that the painter, who was also an asthmatic, said to him, '[name of patient], I've had attacks of coughing and et cetera and I've never been as bad as you. I think we ought to call the ambulance.' He was rushed in, blue light, to resuscitation. I didn't come back because my daughter lives close by and she said, 'Mum, there's nothing you can do and I'm here', et cetera, but he was in there for three or four days. (INTS-PA-009) |
| Support for the patient’s treatment workload seen as an affirmation of the strength of the patient/family member relationship in the face of imminent death | Patient: …my son was like, bless him, he was like, 'Mum, you've got to get your immune system built up', and he was getting me all these different fruits, and making these smoothies and that. I'm thinking oh, my God, I'm getting indigestion, bless his heart. It was all these berries and everything. But bless my son's heart, and my daughter… I couldn't say anything. Because he started going to – [name of supermarket] - and buying these big bags of frozen fruit. They must have cost him a fortune. I'm thinking, oh, gosh, I just don't know how to tell him. I just couldn't stomach another one. But bless him, I mean he was just...  (INTS-PA-010) | Support for the patient’s treatment workload may be seen as an affirmation of the strength of the patient/family member relationship | SYSTEMATIC REVIEW ONLY |
|  |  | Caregivers feel compelled to take on a care-giving role over the long duration of the disease trajectory | Patient: I often feel guilty…I can tell she [patient’s wife] is dying to try and do something and only when I'm really, really bad, I'll say to her, 'Oh, you do whatever you think.' That usually involves phoning up somebody and what have you. I make it difficult for my wife, by not letting her see that I'm actually struggling big time. I'm only struggling a little bit….Up until only recently, I've started to consider [name of wife] and the worries she's having…I'll be feeling like a barrel of shit to be honest. She'll say to me, 'Are you all right?' I say, 'Yes, I'm fine babe.' What help is that to her? How can she respond to that? Actually, when it gets really bad then she does say, 'That's it, I've had enough, I'm calling somebody,' but that's when it gets really bad. (INTS-PA-001) |
|  |  | Delegated workload of treatment tasks that informal carer has to carry out | Patient: [My family] help carry [the oxygen concentrator] around and [daughter’s name] is very adept at switching from this to the full size concentrator which I have in one of the rooms which I normally - but of course the lead won't stretch from here.  Researcher: Do they keep a little eye…out for your flare-ups?  Patient: Oh yes, [daughter’s name] especially watches me like a hawk!  (INTS-PA-003) |
|  |  | Informal carer has to undertake domestic tasks previously undertaken by patient | Patient: I'm very mindful that lots of heavy stuff I can't come and do any more, you know, and I'm very reliant on [name of wife] - bless her - in doing a lot of that heavy work, you know, like mowing the lawn and things like that, that I can't do. (INTS-PA-002) |
| Importance of support from empathetic, trusted HCPs in whom patients have faith | Patient: …I carried on seeing [name of specialist oncology doctor] on three-monthly intervals, right up until my last [treatment]. …[Name of specialist oncology doctor] is very nice. He did, in fact, probably a few months ago, say to me did I want to start going to [name of local district general hospital closest to patient]? I said to him, 'Definitely not.' Definitely no, I know what to expect. He said, 'I hope you don't look at me as being a devil!’ Obviously the wrong thing to say to him! …That's the sort of rapport I built up with him as well. I don't feel there isn't anything I can ask him (INTS-PA-019) | Importance of support from trusted HCPs, especially those with specialist knowledge of COPD | Patient: I do a bit of work at [name of local hospice]. I was in there once and I heard them talking about a doctor coming in to give a talk; so I put my hand up and said, 'Please can I come along?' I went, and that was when I met [name of specialist respiratory doctor], who I thought was absolutely wonderful. Next time I saw my GP, I said, 'Oh, by the way, is there any chance I could be referred to him? - because he specialises in my illness and he might be able to give me a bit more idea on how to manage - and maybe even treatment plan.' The GP's very good as well, and he said, 'Of course.' So he made a referral, and that's when I came to see Dr [name of consultant respiratory physician], who is a fantastic man - person, individual - as well as being, what I think, is a very good doctor. (INTS-PA-005) |
| Less commonly, loss of faith in HCPs | Patient: I haven't had a lot of faith…in certain GPs.  (INTS-PA-010) | Importance of relational continuity with HCPs making access to and navigation of the healthcare system and its institutions easier | Researcher: You've got a specific respiratory consultant, have you, that you…  Patient: Yes, [name of respiratory consultant], he's lovely.  Wife: We just think that, even though it means going to [name of local teaching hospital] for a lot of them, it's just the consistency…  Patient: Continuity of care, and the same very luckily at the GP practice…[Name of GP practice] is famous for actually getting that part right, you have your own GP and 90 per cent of the time you will see your own GP who knows you well. Obviously, if you've got an emergency appointment, then you see someone else if we want to, but as a general practice you have a doctor who you can get to know at a personal level and who can get to know you, and who, in my case, I didn't know, I didn't think they did have house calls any more, but they have been prepared on several occasions, 'Oh right, stay there, be there in half an hour' (INTS-PA-007) |
| Specialist clinicians encourage priorities other than treatment | Specialist oncology doctor: I think that if that scan looked fine and everything’s stable and under control, you know, it’ll be post holidays and you may want to then go and have a holiday because that will be a nice time to go  [general laughter]  Specialist oncology doctor: I’m not trying to be a travel agent but, you know,  Husband: No commission on is there?  Specialist oncology doctor: Well, I may have some brochures next door  [general laughter]  Specialist oncology doctor: [suddenly serious] But I think it’s the right thing to do, you know. I think the chemo was harder than you let on, I think. And I think I pushed you quite hard because it seemed to be doing you a lot of good but I just think you probably got as much good out of it as you could get…and I think that it’s probably the right time to have a little rest from it (OB-PA-042) | Loss of faith in healthcare professionals | INTS-PA-006: There was a lady in a wheelchair with an oxygen cylinder about the same age as me….She was pulling her oxygen cylinder behind her when we're doing the walk at the end. I remember thinking, no, I'm not going there, I have to do something about this. Okay? I started reading up and looking on YouTube. I think what disappointed me, we had 12 sessions and only one session on diet [at pulmonary rehabilitation]. The other 11 were on physical activity. It also made me feel was, it was really all about managing your symptoms, not trying to better your symptoms. Basically they were expecting us to have lots of exacerbations, going to hospital when it got really bad, and I'm thinking no, this is not good enough. Really from that first time I went, which is - I've looked elsewhere for treatments, okay? (INTS-PA-006) |
| Flexible and responsive treatment experience | Doctor: Perfect. So that means erm, and let me get this right, [days] are good?  Wife: Yes. [days] I don’t look after a lot of other people, yes it is good.  Doctor: Fine. So obviously go ahead next week and then if I see you guys three weeks from today  Patient: Three weeks from today  Doctor: Another set of bloods on the day. Then it’ll save you having to faff around with taxis and all of that won’t it? (OBS-PA-030)  Patient: [name of lung cancer CNS]…I can get in touch with…and she's been very helpful. If anything I'm not sure, I'll just phone [name of lung cancer CNS] and she deals with it for me (INTS-PA-015) | Knowledge and skills gained from specialist care vital | Patient: [pulmonary rehabilitation was a] real major turning point because apart from actually getting me working a little bit, not very much but a bit, I learned a lot more about COPD and that was very important… because I started going forward (INTS-PA-009) |
|  |  | Inflexibility of treatment experience | Specialist respiratory doctor: Have you managed to get on the rehab thing at all?  Patient: I think January I’ve got an appointment er…assessment  Specialist respiratory doctor: That’s really important…that’s really good news um. …  Patient: Cos she rung me up…it was either um I think it was this Friday [sound of computer clicking] or…but until the last moment I couldn’t get an appointment…I couldn’t get me holidays off…I’ve got to sort my working week out now.  Specialist respiratory doctor: You do need to do that  Patient: Because it [rehab] is Monday and Friday.  (OBS-PA-010) |
| Little peer support available for patients with lung cancer. What is available appears impromptu and transitory | Patient: I know of a couple of people, and I can pick up the phone to them… every so often we'll catch up on the phone, or we'll try and meet up for a coffee or something.  Researcher: Did you meet them at the hospital?  Patient: I have done, but they're not on the same treatment as me. They were on chemotherapy and then went on to have radiotherapy, and that's all stopped. So now and again I might bump into them when they have their three-month check-up…so, yes, I sometimes bump into them then.  (INTS-PA-010) | Peer support is an important resource and is generally accessed through pulmonary rehabilitation | Patient: I had more difficulty [doing pulmonary rehabilitation (PR) exercises] and it was not just me, there was another lassie that goes called [name of peer]. I was really glad that she was there because we seemed to have difficulties on the same days. Again, we sat and thought about this: What's going on? Why can't we do it? Then you do one day and another and we found atmospherics and, you know…because I said to [name of peer], 'I don't know what's going on; I found it really, really hard today.' 'So did I.' 'Ah, right.… I've got a friend called [name of peer] and a friend called [name of peer] and we were together earlier. We are there for each other…It rather shames me when I'm feeling like: Oh, I don't really want to do this - and I look and I think: You will! You will!  (INTS-PA-005) |
|  |  | Shared experiences with peers reduces isolation | Researcher: So having that peer, it almost pushes you, you find?  Patient: Yes, motivates you and stops you feeling sorry for yourself and you get on with it… having these two pals is helpful, very helpful; we lift each other up and commiserate and laugh and just generally help each other. (INTS-PA-005) |
|  |  | Peer support is used as a resource for information sharing | Patient: …one of the things that I noticed was different people [at pulmonary rehabilitation], people who were on oxygen, they had different pieces of equipment, and some of them weren't too good [laughs] and people used to ask me about that. Yes, so I suppose it was good because I was able to give some people information about what else was available and what they could do.  (INTS-PA-003) |
| Short disease trajectory: ill equipped to self-manage symptoms at home | SYSTEMATIC REVIEW ONLY | Long disease trajectory: get to know their bodies and symptoms, through trial and error | Patient: I took control over the specialist with that because when I listened to them they've landed me back in hospital…I said to [the specialists] I'm listening to my body now and I'm going to go by my body with needs for medication, everything…I did cut a lot of my medication out because what I used to do was cut it down and see if I could manage it. If I felt I didn't need it, wouldn't take it. I know it's sometimes a bit naughty but it worked….I know you're supposed to take your tablets all separate; I have so many - well, it's like a chemist….I take them all together and I got so fed up of swallowing tablets every time I ate anything that I argued with the nurse about the diabetic stuff and she gives me 1,000 milligrams in the morning and 1,000 milligrams in the evening, plus the insulin so that I can have a rest in the mid-day; I can go out and not worry about any tablets whatsoever, just my insulin. But I take my tablets in the morning and I take it with - I don't swallow them with water, I put them all in my mouth and swallow them with my breakfast!  (INTS-PA-013) |
| Patients are considered culpable for their illness and stigmatized by society | SYSTEMATIC REVIEW ONLY | Patients are considered culpable for their illness and stigmatized by society | Patient: Oh yes, and the thing that amazed me, I guess, at the very beginning, when it was first diagnosed, and it was, you know, 'Do you smoke?' Obviously, I mean, COPD is very smoking related but it's not. Smoking is just another aspect of it, but everyone assumes, 'Oh he's a smoker.'… people around me. If they saw you out of breath, 'Oh you ought to give up the fags,' and all of these sorts of things. Initially I thought it was just the fags, you know, I was totally unaware it was anything else.  (INTS-PA-002) |
| Patients consider themselves culpable for their illness: a “self-inflicted” disease | SYSTEMATIC REVIEW ONLY | Patients consider themselves culpable for their illness: a “self-inflicted” disease | Patient: Plus years of smoking I expect didn’t help [in relation to respiratory symptoms].  Specialist respiratory nurse: No, I don’t think so.  [patient laughs]  Specialist respiratory nurse: Might have a slight part to play mightn’t it?  Pt: Well, I didn’t listen though, did I?  Specialist respiratory nurse: But you’re not smoking now?  Pt: No, no. 4 years now. Yeah but, I’ve been ill ever since I’ve packed up.  Specialist respiratory nurse: Yeah and that’s sometimes…smokers hear that and don’t want to give up because they think oh I’m just going to get ill.  Pt: If I’d have known I wouldn’t have given up… well, no if I hadn’t given up, I’d be dead by now yeah so.  Specialist respiratory nurse: Well, I can’t predict the future but you may well be in a worse situation if you hadn’t given up smoking  Pt: I’ve got no intention of going back.  (OBS-PA-002) |
| Patients experience ‘felt’ stigma of blame, guilt and shame | SYSTEMATIC REVIEW ONLY | Patients experience ‘felt’ stigma of blame, guilt and shame | Patient…I was then told I was suffering from COPD. It's smoking related - I presume, anyway. I remember being quite shocked, and ashamed to a degree. I think this is very much an element of people with COPD that have been smokers - self-blame, you know, and not expecting any sympathy, really… (INTS-PA-005) |
| Patients attempt to conceal their condition owing to fear of ‘enacted’ stigma leading to social isolation | SYSTEMATIC REVIEW ONLY | Patients attempt to conceal their condition owing to fear of ‘enacted’ stigma leading to social isolation | Patient: …The other thing is, I actually do still suffer from embarrassment of what my complaint is…as I explained to somebody the other day, everybody has heard of the big C, cancer, if you say cancer everybody, 'Oh, terrible,' yes. If you've got one leg missing people can easily see it…I've got a blue badge, my wife parks in a disabled bay, she's all right because she's got more brass neck than I have. I actually feel guilty getting out, because when I get out of the car people look at me and go, 'Two arms, two legs, two eyes, doesn't look as if he's struggling. No walking stick, no - why is he parked there?' What they don't realise is me walking from that car to the hole in the wall and back again, by the time I've done that my chest is boom, like that, and I - but they don't see that, all they're seeing is, 'Well he doesn't look as if he's old, he doesn't look like he's disabled. Why the hell is he doing that?' Whereas if somebody says, 'Well, he's got cancer,' they all go, 'Oh, well that's fine, ah yes, that makes sense.' Maybe COPD and pulmonary disease isn't, things like that, need to be, I was going to say advertised but that's not…it needs to be made, people need to be made more aware of it. (INTS-PA-012) |
| Patients feel ‘marked’ by visible treatment leading to social isolation | SYSTEMATIC REVIEW ONLY | Patients feel ‘marked’ by visible treatment leading to social isolation | Patient: I must admit when I first started to go out with this there was a very big embarrassment about it [oxygen]. I didn't want to use it. I carried it, but I never put the thing on…you carry it in a carrier bag so it's not necessarily so visibly obvious….We went shopping in [name of local town] somewhere and we were sat down having a bite to eat for lunch, [name of wife] went off to get what we were eating, and I was sat on the chair and I saw a little girl with her mum suddenly come along and I knew what the little girl… She looked at me and I knew exactly what she was going to say to her mum before she even said it, you know, 'Mummy that man there's got something on his nose,' and of course mum turned around and realised and smiled and obviously I smiled back and said, 'It's not a problem'. (INTS-PA-002) |
|  |  | Patients internalise stigma, considering themselves undeserving of treatment | Patient: Er no, because every time you’ve explained most of it, it’s just me that’s been lacking… holding everything up by smoking  Specialist respiratory doctor: I’m not that, well I’m not in that, I’m not in that whatever. To be honest with you, that’s in the past. Move forward  Patient: Like I say, it was all there for me in the past. And as I say just…  (OBS-PA-010) |
|  |  | Patients experience ‘enacted’ stigma from HCPs, making access to treatment challenging | Patient: I had a bit more extensive x-ray. I think I had an MRI, didn't I, and so on? They started talking about COPD, which my GP explained. 'There's lots of cilia getting burnt off and that's because you've been smoking, you silly fool', et cetera. (INTS-PA-009) |
| Embarrassment about symptoms, medications and treatment technologies which mark the patient as ill leading to fear of ‘enacted’ stigma | SYSTEMATIC REVIEW ONLY | Embarrassment about symptoms, medications and treatment technologies which mark the patient as ill leading to fear of ‘enacted’ stigma | Patient: I don't use mobile phones as such, but I've got a mobile phone for the simple reason…it took the place of what I'd started doing, which was window shopping, which is - everybody's so used to seeing people walking down the street and then stopping and going, I used to think oh no, wait, actually if I get hard of breathing, I can take my mobile phone out and I can stand there and I can go like that. I can be typing away, I write a load of rubbish as well, but nobody knows that. People will just wander by and think there's a bloke on his mobile phone texting somebody. Embarrassment gone away, nobody knows any different… I'd rather people ignored me than stop and go, 'Are you okay? Can I do something?'…That makes me even worse, because then I start getting, 'Yes, yes, I'm fine, yes, just go away, leave me alone.' (INTS-PA-012) |
|  |  | Exacerbation triggers – leads to avoidance of social situations | Patient: Touch wood, you know touch wood I haven't touched any antibiotics for now, it must be a year-and-a-half.  Wife: About a year I'd say, yes, but I think the main thing for that very, very sadly is to isolate ourselves and that is tough, and people don't really talk about it. They say, especially with [name of patient]’s prognosis, you have to get out there and you have to live, but the problem is, in winter especially, in doing that it could actually kill you. You haven't really been out during the winter months at all, certainly this winter, and where I'm not at work any more and we're not handling paperwork or the same materials et cetera, and I've also had to restrict my social activities during winter.  Patient: Yes.  Researcher: Because of the risk of infection?  Wife: Absolutely, I'm paranoid with hand sanitizer. You can buy stuff, whether it works or not, but it seems to have worked perhaps, Cold Guard around your nose and how you touch, so just to be very, very aware, very aware of people around you. If they have colds, you don't go and see them. That has been, I think, psychologically on both of us, extraordinarily tough  (INTS-PA-007) |
| Illness as contagious: social networks contract as friends withdraw | Patient: Yeah, too many horror films. It’s like saying, you know. You’d be surprised how many people I get the impression that they shouldn’t get too close to you if you’ve got or had cancer. They get the impression that it’s going to…  Specialist oncology doctor: They think it might be catching. I’m not going to have a very long life then am I?  [general laughter]  Patient: You’ve had it! You shouldn’t be here now [laughs]  (OBS-PA-035) | Illness as contagious: social networks contract as friends withdraw. Isolation worsens with disease progression and deterioration of physical function | Wife: We had been going through - well, *I* had been going through hell, to be quite honest, because 'I'm going to die. I'm going to die….No, you're not. No. You're fine.' In the streets [gasp] and people stopping and saying, 'Can we help?' 'No, no, no.'  Patient: Thinking you're drunk.  Researcher: Did it stop you going out?  Patient: Yes.  Wife: Yes.  Patient: I still don't like going out on my own. In fact, it's a very rare event even now. I haven't got used to it.  Wife: You *never* go out on your own.  Researcher: Is that because - why is that?  Patient: Nervous about making too much of a fool of myself. Some of these attacks have unwanted side-effects, one of which is to lose control, continence, and that's pretty horrible and that's happened several times and you know, sort of pads and all that sort of thing… Well, it's not nice. Heaven's above you know, I played rugby, you know [laughs]. All that sort of thing is a bit, well, to me, very degrading (INTS-PA-009) |
| Psychological co-morbidities lead to avoidance of social situations | SYSTEMATIC REVIEW ONLY | Logistical difficulties of treatment workload limits patient to home | Specialist respiratory nurse: Have you ever thought about getting a little walker or something?  Patient: Um. Well I have one of them well you know. I have got one actually.  Specialist respiratory nurse: You can put your oxygen in that possibly.  Patient: It used to fill up all the room in the bus. I used to feel all you know sort of  Specialist respiratory nurse: I wouldn’t worry about it. You’re perfectly entitled…  Patient: There’s all these yummy mummies with their prams and me with my walker [laughs].  Specialist respiratory nurse: I expect they feel the same though. They probably feel that they take up a lot of room with the prams.  Patient: Nah, nah.  (OBS-PA-002) |
|  |  | Social isolation extends beyond patient to affect informal caregiver | Wife: I think the main thing for that very, very sadly is to isolate ourselves and that is tough, and people don't really talk about it. …I've also had to restrict my social activities during winter….[you have] to be very, very aware, very aware of people around you. If they have colds, you don't go and see them. That has been, I think, psychologically on both of us, extraordinarily tough  (INTS-PA-007) |
|  |  | Psychological co-morbidities lead to avoidance of social situations | SYSTEMATIC REVIEW ONLY |
